# Social frailty as a predictor of all-cause mortality and functional disability: a systematic review and meta-analysis

**DOI:** 10.1038/s41598-024-53984-3

**Published:** 2024-02-10

**Authors:** Takaharu Goto, Takahiro Kishimoto, Shinji Fujiwara, Yasuhiko Shirayama, Tetsuo Ichikawa

**Affiliations:** 1 Department of Prosthodontics and Oral Rehabilitation, Tokushima University Graduate School of Biomedical Sciences, Tokushima, Japan; 2 Department of Comprehensive Dentistry, Tokushima University Graduate School of Biomedical Sciences, Tokushima, Japan; 3 Mima Municipal Koyadaira Clinic, Tokushima, Japan; 4 Department of Oral Health Science and Social Welfare, Tokushima University Graduate School of Biomedical Sciences, Tokushima, Japan

**Keywords:** Medical research, Risk factors

## Abstract

The association between social frailty and adverse health outcomes, especially mortality and functional disability, which are essential health outcomes, has not been systematically summarized or meta-analyzed. In this study, we conducted a systematic review and meta-analysis of the impact of social frailty on all-cause mortality and functional disability, while addressing the components of social frailty. In this study, social frailty was operationally defined in alignment with the previous literature, as follows: “a state of increased vulnerability to the interactive back-and-forth of the community, including general resources, social resources, social behaviors, and needs.” Hazard ratios or odds ratios described in each selected literature were used as the meta-analytic results. Considering the impact of social frailty on all-cause mortality, the hazard ratio was 1.96 (95% CI 1.20–3.19), indicating a significant association between the two but high heterogeneity. The hazard and odds ratios for the impact of social frailty on functional disability were 1.43 (95% CI 1.20–1.69) and 2.06 (95% CI 1.55–2.74), respectively. A significant association was found between social frailty and functional disability; both hazard and odds ratios were found, and low heterogeneity between these articles was observed. These results highlight the importance of assessing social frailty using more standardized methods and examining its effects on various health outcomes.

## Introduction

The older adult population is increasing worldwide owing to the increasing life expectancy and declining birth rates^1–3^. The aging rate, which is the percentage of the older population aged 65 years and above in the total population, is projected to rise from 8.3% in 2015 to 18.1% by 2060, and the population is expected to age rapidly over the next half century, posing a great challenge for both developed and developing nations^4–6^. Against the background of this increase in the older adult population, the concept of frailty, which indicates an increased vulnerability to various health problems attributed to decreased physiological reserves associated with aging, has been widely employed^6–9^. The accumulated deficit model evaluates frailty from multiple perspectives, including physical function, and frailty is sometimes described as physical, psychological, or social^10,11^. Physical frailty has received the greatest amount of attention, with research demonstrating its adverse impact on health, including morbidity and mortality^12–16^. However, more research is necessary on other aspects of frailty, such as psychological and social frailty.

In this context, social frailty can be defined in alignment with previous literature, as follows: “a state of increased vulnerability to the interactive back-and-forth of community including general resources, social resources, social behaviors, and needs.” Social frailty has recently attracted much attention and has been shown to precede physical frailty^17^. For example, social isolation, considered one of the components of social frailty, has an impact on various physical and psychological aspects, resulting in cognitive decline, depressive symptoms, poor sleep quality, and reduced levels of physical activity^18–20^. Bunt et al.^21^ reported that social frailty could be categorized into four components, as follows: general resources, such as living alone or financial difficulties; social resources, such as the presence of either friends or neighbors or both or a confidant; social behaviors, such as social participation or volunteering; and the fulfillment of basic social needs, such as social loneliness or social support. These components are affected by the risk factors of aging. For example, an individual might lose their spouse when becoming older, and find it challenging to participate in social activities to the same extent as before because of feeling lonely in a crowded room. Therefore, social frailty is a crucial issue to consider in the current context of a growing older population. However, the association between social frailty and adverse health outcomes, especially all-cause mortality and functional disability, which are essential health outcomes, has not been systematically summarized or meta-analyzed.

In this study, we conducted a systematic review and meta-analysis of the impact of social frailty on two health outcomes, all-cause mortality and functional disability, while addressing the components of social frailty.

## Results

**Figure 1:**
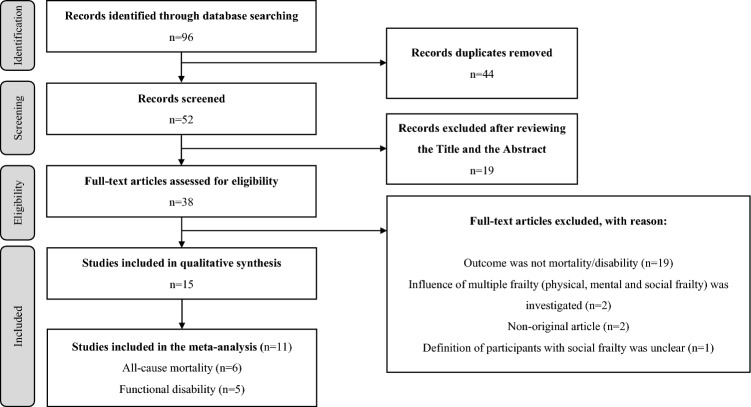
Literature review strategy.

The literature review strategy is illustrated in Fig. 1. After the primary search and screening, 52 articles were retrieved. After screening the titles and abstracts, 38 articles that met the purpose of this study were reviewed in their entirety. Finally, 15 articles were selected for qualitative synthesis of all-cause mortality and functional disability. Subsequently, a meta-analysis was performed on 11 of these 15 articles, for which either hazard or odds ratios were calculated.

### Results of qualitative synthesis

Table 1 summarizes the six articles with all-cause mortality outcomes and one with all-cause mortality or functional disability outcomes^22–28^. Articles on all-cause mortality outcomes were reported from 2013 to 2022, including five prospective cohort studies and one retrospective cohort study. Prospective cohort studies varied in follow-up time from 12 to 96 months, and all articles calculated the hazard ratios for all-cause mortality. Participants in these studies were community-dwelling older individuals. Jujo et al.^25^ and Ono et al.^26^ studied patients with heart failure and gastrointestinal cancer, respectively, whereas Adachi et al.^27^ studied hospitalized patients with cardiovascular disease. Considering the impact of social frailty on all-cause mortality, four of the six articles reported a significant association, whereas two articles concluded that there was no significant association.

**Table 1:** Characteristics of the included studies for the outcome of “all-cause mortality” and “all-cause mortality or functional disability”.

References	Year	Study design	Follow-up duration	Location	Sample size/characteristics	Results	Quality
All-cause mortality							
Garre-Olmo et al.^22^*	2013	Prospect	48 months	Spain	875/366 males and 509 females; age, 81.7 ± 4.8 years	The risk of mortality was higher in those experiencing SF (HR: 2.69).	6
Ma et al.^23^*	2018	Prospect	96 months	China	1697/aged ≥ 60 years	After adjusting for age and sex, the risk of 8-year mortality was higher in those experiencing SF (HR: 3.273).	6
Gobbens et al.^24^*	2021	Prospect	84 months	Neatherlands	479/207 males and 272 females; age, 80.3 ± 3.8 years	The risk of mortality was not significantly higher in those experiencing SF after adjustment for age and gender (HR: 1.17).	7
Jujo et al.^25^*	2021	Prospect	12 months	Japan	1240/Patients with heart failure; 713 males and 527 females	The risk of mortality was higher in those experiencing SF, even after adjusting for key clinical risk factors (HR: 1.53).	8
Ono et al.^26^*	2021	Prospect	53 months	Japan	181/Patients with GC; 139 males and 42 females; age, 72.0 years	The risk of mortality in patients with GC was higher in those experiencing SF after adjusting for confounding factors (HR, 3.23).	7
Adachi et al.^27^*	2022	Retro	12 months	Japan	184/Hospitalized elderly patients with CVD; 122 males and 62 females; age, 75.0 years	The risk of all-cause clinical events, including all-cause mortality was not significantly higher in those experiencing SF (HR: 1.41).	7
All-cause mortality or functional disability							
Yamada and Arai^28^	2018	Prospect	72 months	Japan	6603/2911 males and 3692 females; age, 75.2 ± 6.6 years	The risk of incident disability and all-cause mortality was higher in those experiencing SF (HR: 1.28).	6

*SF* social frailty; *HR* hazard ratios; *Prospect* prospective study; *Retro* retrospective study; *GC* gastrointestinal cancer; *CVD* cardiovascular disease.

*Articles included for meta-analysis.

Yamada et al.^28^ reported an association between social frailty and all-cause mortality or functional disability as a health outcome. This prospective cohort study of 6,603 community-dwelling older adults reported that social frailty was a significant risk factor for all-cause mortality and functional disability.

A summary of the eight articles on functional disability outcomes is presented in Table 2^29–35^. Articles reporting outcomes of functional disability were published between 2014 and 2022, with four reports each from prospective cohort and cross-sectional studies. Regarding certification or definition of the onset of functional disability, the use of the new long-term care insurance service and the need for assistance with ADL or instrumental ADL (IADL) items were assessed. Certain studies assessed the degree of functional disability using the Katz IADL^29^, Lawton IADL^34^, and Groningen Activity Restriction scales^30^. The duration of follow-up of the prospective cohort studies ranged from 12 to 60 months. Teo et al.^32^ reported cross-sectional and prospective cohort studies in the same article, considered separate reports in the present study. Of the eight reports, only Ament et al.^29^ concluded that social frailty did not affect the occurrence of IADL impairment, whereas the other seven articles reported a significant association between social frailty and ADL or IADL impairment. Teo et al.^32^ and Usui et al.^34^ classified the participants according to their physical frailty status and examined the impact of social frailty.

**Table 2:** Characteristics of included studies for the outcome of functional disability.

Author	Year	Study design	Follow-up duration	Location	Sample size/characteristics	Results	Quality
Ament et al.^29^	2014	Prospect	12 months	Netherlands	334/134 males and 200 females; age, 78.1 ± 4.9 years	Author did not find an effect for SF on IADL disability.	4
Gobbens et al.^30^	2015	Cross-sec	–	Netherlands	221/81 males and 140 females; age, 84.8 ± 8.9 years	SF correlated only with disability (CC, 0.19).	7
Makizako et al.^31^*	2015	Prospect	24 months	Japan	4304/2097 males and 2207 females; age, 71.7 ± 5.3 years	The risk of disability was higher in those experiencing SF (HR: 1.66).	8
Teo et al.^32^*	2017	Cross-sec	–	Singapore	2406 / 882 males and 1524 females; age, 66.1 ± 7.6 years	Pre-frail or frail with SF significantly increased the odds of disabilities (OR: 1.96).	7
Teo et al.^32^*	2017	Prospect	36 months	Singapore	1254	Pre-frail or frail with SF significantly increased the odds of disabilities (OR: 2.07).	7
Park et al.^33^*	2019	Cross-sec	–	Korea	408/172 males and 236 females; age, 74.9 ± 6.0 years	SF significantly increased the odds of disabilities impacting ADLs (OR: 2.53).	8
Usui et al.^34^	2021	Cross-sec	–	Japan	158/113 males and 45 females; age, 74.1 ± 6.8 years	In the non-physical frailty group, the number of IADL disabilities was significantly higher in patients with SF than in those without.	6
Doi et al.^35^*	2022	Prospect	60 months	Japan	4642/2312 males and 2330 females; age, 71.7 ± 5.3 years	The risk of disability adjusted for covariates was higher in those experiencing SF (HR: 1.40).	7

*SF* social frailty; *Prospect* prospective study; *Cross-sec* cross-sectional study; *HR* hazard ratios; *OR* odds ratio; *ADL* activity of daily living; *IADL* instrumental activities of daily living; *CC* correlation coeffecience.

*Articles included for meta-analysis.

Considering the age of the participants, Gobbens et al.^30^, with functional disability as a health outcome, included participants aged 51 and older (mean age, 84.8 years), while all other studies included participants aged at least 60 years. Regarding the social frailty assessment tools used across all included studies, the Makizako index was the most frequently used tool in seven reports, followed by the Tilburg Frailty Indicator (TFI) and Social Frailty Index (SFI) in two reports each. In one report each, the Yamada index, Groningen Frailty Indicator (GFI), Garre-Olmo index, and help, participation, loneliness, financial, and talk scales (HALFT) were used. Regarding confounders, age was adjusted in five out of six reports on all-cause mortality and seven out of eight reports on functional disability. Furthermore, disease severity and physical factors other than age and sex were adjusted in three out of six reports on all-cause mortality and six out of eight reports on functional disability. Representative confounders were clinical laboratory values such as blood pressure, body mass index (BMI), and medication status, as well as the Meta-analysis Global Group in Chronic Heart Failure (MAGGIC) risk score^25^ (for all-cause mortality in patients with heart failure) and the Union for International Cancer Control (UICC) stage categories^26^ (for lung cancer), depression^31,32^, cognitive function^31,32,35^, and degree of physical frailty^31,32,35^.

### Methodological quality of the studies

Tables 1 and 2 present the quality of the selected articles. Ten of the 11 longitudinal studies assessed using the Newcastle–Ottawa Scale were of high quality. Only one study^29^ reported a quality score of 4 because the outcome of functional disability was assessed by the degree of IADL in the selection section, which may have included certain participants with disabilities at the beginning of the study (baseline), and the confounding factors were not adjusted for comparison. Among the four cross-sectional studies assessed by the Agency for Healthcare Research and Quality (AHRQ), two studies each were of moderate quality and high quality. The quality assessment for each study is shown in Supplementary File S1.

### Meta-analysis result of all-cause mortality

**Figure 2:**
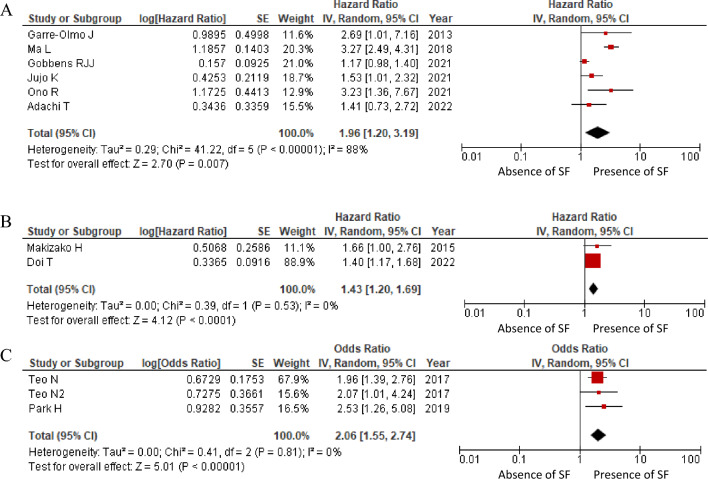
Forest plot evaluating the effects of the presence or absence of social frailty. (**A**) hazard ratio of all-cause mortality; (**B**) hazard ratio of functional disability; and (**C**) odds ratio of functional disability.

The results of the all-cause mortality meta-analysis are shown in Fig. 2A. Since all six reports^22–27^ that were qualitatively integrated in all-cause mortality had hazard ratios, we attempted to integrate the meta-analyses of these reports quantitatively. Considering the impact of social frailty on all-cause mortality, the hazard ratio was 1.96 (95% CI 1.20–3.19), indicating a significant association between the two but high heterogeneity (I^2^ = 88%, *p* < 0.05). The fixed-effects model was used as a sensitivity analysis, obtaining a similar result with a hazard ratio of 1.63 (95% CI 1.42–1.86).

### Meta-analysis result of functional disability

The results of the meta-analysis on functional disability are shown in Fig. 2B,C. Of the eight reports^29–35^ that were qualitatively integrated on functional disability, two^31,35^ and three reports^32,33^ calculated hazard and odds ratios, respectively. Therefore, we attempted to integrate these reports quantitatively by meta-analysis. The hazard and odds ratios for the impact of social frailty on functional disability were 1·43 (95% CI 1.20–1.69) and 2.06 (95% CI 1.55–2.74), respectively. A significant association was found between social frailty and functional disability; both hazard and odds ratios were found, and low heterogeneity between these articles was observed (I^2^ = 0.0%, *p* < 0.05).

## Discussion

In the accumulated deficit model, physical frailty, including physical activity and nutritional intake, has been associated with various health outcomes since early times, and certain reference values for physical frailty have been established, as reported by Fried et al.^36^. However, social frailty in the accumulated deficit model has also attracted attention. Previous reports on the prevalence of social frailty in community-dwelling older adults reported rates ranging from 8.4 to 11.1%^17,22,31^. Considering the results of a meta-analysis that showed a prevalence of physical frailty of 9.9%^37^, these rates are comparable or slightly higher. Discussion of social frailty is essential owing to the ever-increasing older adult population worldwide. The relationship between social frailty and various health outcomes has been reported. Pek et al.^38^ reported that social frailty is significantly associated with the risk of reduced physical performance, physical inactivity, depressive symptoms, and malnutrition. Hironaka et al.^39^ reported that social frailty was significantly associated not only with physical frailty, but also with oral frailty, such as chewing ability and tongue pressure. Other associations between quality of life and hearing loss have also been reported^40–42^. However, the results of these studies were inconsistent. Moreover, there are no reports integrated by meta-analyses. In this study, the authors conducted a meta-analysis focusing on the health outcomes of all-cause mortality and functional disability to better clarify social frailty. Based on the available evidence, this is the first meta-analysis on social frailty.

The results of the functional disability meta-analysis were significant for both hazard and odds ratios, and low heterogeneity was observed between the included studies. This result can be attributed to the fact that all the studies included in the meta-analysis reported a significant association between social frailty and the occurrence of ADL or IADL impairment; that is, the results were consistent. Considering the assessment tools for social frailty, the results of the hazard ratios were nearly constant since both reports used the Makizako index, and the results of the odds ratios were also nearly consistent since two reports used the SFI, and one report used the Makizako index. Considering the impact of social frailty on functional disability, social frailty is significantly associated with reduced physical activity and muscle strength^21,43^. Pothier et al.^44^ also reported that inflammatory markers, such as C-reactive protein and interleukin-6, are associated with social frailty. The results of these studies justify the findings of our meta-analysis.

On the contrary, although the results of the meta-analysis of the impact of social frailty on all-cause mortality showed high heterogeneity, the hazard ratios were significant. High heterogeneity could weaken the evidence for the results obtained in the meta-analysis, and caution should be exercised when interpreting these results. This high heterogeneity can be explained by the fact that four of the six reports on all-cause mortality showed significant associations, while two reports, those of Gobbens et al.^24^ and Adachi et al.^27^, did not show significant associations; the results were inconsistent. This inconsistency could be attributed to the variation in the assessment tools for social frailty in the studies: three of the six reports used the Makizako index, and one report each used the TFI, Garre-Olmo's index, and the HALFT scale. Each assessment tool also has different question items, number of questions, and cut-off values. A subgroup analysis of the three reports^25–27^ using the Makizako index showed a hazard ratio of 1.71 (95% CI 1.15–2.56), which was as significant as that of 1.96 (95% CI 1.20–3.19) when all six reports were included, while heterogeneity was reduced (I^2^ = 25%; *p* < 0.05, Supplement File S2). The Makizako index consists of the following five components: living alone, going out less frequently compared with last year, visiting friends, feeling helpful toward friends or family, and talking with someone every day; in other words, these components indicate social activities, social roles, and social connections. Participants are classified as socially frail if their total score is 2 or higher. However, on the HALFT scale, financial difficulties are included as a component of social frailty, and the content differs from one assessment tool to another. As mentioned above, the results of the meta-analysis on all-cause mortality showed a high degree of heterogeneity. However, it is also significant that almost all reports were adjusted for confounding factors; the quality of the included articles was high, and the hazard ratio of social frailty on all-cause mortality was 1.96.

Following the scoping review by Bunt et al.^21^, we defined social frailty as "a state of increased vulnerability to the interactive back-and-forth of community including general resources, social resources, social behaviors, and needs.” Bunt et al. suggested that these four components of social frailty are mutually influenced, which supports the idea that the symptoms of aging are a combination of various factors rather than being isolated ones. This combination justifies the difficulty of assessing social frailty. Although the methods used to assess social frailty in the selected papers were within the scope of our definition, a more robust definition of social frailty and a standardized questionnaire/assessment of social frailty with reliability and validity must be developed. In addition to psychosocial and subjective measures, such as loneliness and emptiness, it is also essential to use more objective measures of social frailty, such as living alone and frequency of outings. The results show that the meta-analysis of functional disability was consistent, while that of all-cause mortality was heterogeneous, which may indicate that social frailty precedes physical frailty.

The strength of this study is that the review was conducted using the methods recommended in the PRISMA statement, and the meta-analysis was conducted considering the quality of the article. The main limitation of this review was that the number of articles available for the meta-analysis was somewhat limited, as follows: six for all-cause mortality and two each for the hazard and odds ratios for functional disability. In particular, for functional disability, the I^2^ values were low, but the number of selected studies was negligible. It is known that the I^2^ value, a statistic that indicates heterogeneity, is affected by the number of studies selected. Some reports suggest that if the number of studies is too small, the calculated value does not reflect the actual amount of heterogeneity^45^. Therefore, the interpretation of the results of this study on functional disability requires caution, and meta-analyses with more literature are recommended in the future. Another limitation of this meta-analysis was that examining publication bias using funnel plots was not possible because fewer than 10 reports were available. Furthermore, as mentioned above, the high degree of heterogeneity with respect to all-cause mortality was also a limitation. However, we believe that this study is significant because it confirms current evidence of the impact of social frailty on health outcomes, such as all-cause mortality and functional disability, regarding hazard ratios, odds ratios, and heterogeneity. Future long-term longitudinal studies are warranted to assess social frailty using more standardized methods and examine its association with various health outcomes. In this study, social frailty was operationally defined in alignment with the previous literature, as follows: “a state of increased vulnerability to the interactive back-and-forth of the community, including general resources, social resources, social behaviors, and needs.” Social frailty encompasses numerous components and confounding factors, making it challenging to determine currently which assessment items of social frailty critically impact functional disability or all-cause mortality. Further discussion of social frailty should explore its definitive components, considering individual factors such as age, sex, race, and various environmental factors.

## Conclusions

This systematic review and meta-analysis demonstrated that social frailty was significantly associated with all-cause mortality and functional disability regarding hazard and odds ratios. However, high heterogeneity was found in the included studies concerning all-cause mortality. These results highlight the importance of assessing social frailty using more standardized methods and examining its effects on various health outcomes.

## Methods

This systematic review and meta-analysis were conducted in accordance with the Preferred Reporting Items for Systematic Reviews and Meta-analyses (PRISMA) statement^46^. The study was registered as a protocol in the International Prospective Register of Systematic Reviews (PROSPERO #CRD42023397856).

### Operational definition of social frailty in this study

Considering the principle that frailty can be modifiable by appropriate interventions and comprehensive views that have been reported in the past, social frailty is defined here as “a state of increased vulnerability to the interactive back-and-forth of community including general resources, social resources, social behaviors, and needs.”

### Search strategy

We searched the searched MEDLINE (via PubMed), Cochrane Library (via the Cochrane Central Register of Controlled Trials, CENTRAL), and Scopus databases for studies published between January 1, 1980, and December 31, 2022. Combinations of the following Medical Subject Heading (MeSH) terms and text terms were used: [(“mortality”) OR (“mortality” [Mesh]) OR (“disability”)] AND (“social frailty”). In addition to these database searches, manual searches were performed. The searches were conducted individually by two authors (T. G. and T. K.) who had previously confirmed the literature search criteria.

### Eligibility criteria

The following selection criteria were used to select the literature for this review: (1) articles that clearly defined individuals with social frailty, (2) articles that described the relationship between the primary outcome (all-cause mortality and functional disability) and social frailty, (3) articles that described the impact of social frailty alone, not multiple frailties; and (4) original papers written in English. Articles that did not meet the inclusion criteria were excluded. The articles included in the meta-analysis were drawn from articles that met the above inclusion criteria. These articles were required to have a hazard or odds ratio with a 95% confidence interval (CI) to facilitate appropriate meta-analytic procedures.

### Outcomes

The primary outcomes of this study were all-cause mortality and functional disability rates. The outcomes reported in this article are estimates of the longest possible follow-up period. The onset of functional disability was determined based, for example, on the use of a new long-term care insurance service or the decline in activities of daily living (ADL). The assessment tools used for the certification or definition of functional disability were confirmed by the descriptions in the text.

### Study selection and data extraction

After excluding duplicate articles from the databases, articles that adhered to the purpose and selection criteria of this study were selected from the titles and abstracts. After excluding articles irrelevant to the content of this study, full text screening was performed, and the articles were extracted again. When the results of the two examiners did not match, a discussion was held with a third author (SF), and the final selection of articles was made.

An extraction sheet was created for data collection using Microsoft Excel software (Microsoft Office Professional 2016, CA, USA). The sheet included the article number, author, year of publication, reporting institution, country, study design, sample size, definition of the participants with social frailty, results, and article quality. The results were summarized on the basis of the primary outcome.

### Quality assessment

The Newcastle–Ottawa Scale^47^ for longitudinal studies and the Agency for Healthcare Research and Quality^48^ for cross-sectional studies were used to assess article quality. The Newcastle–Ottawa Scale consists of three qualitative parts with nine items, as follows: selection (four items), comparability (two items), and outcome/exposure (three items). In this study, a score ≥ 5 indicated high quality^47^. The AHRQ is an 11-item checklist with a score of 1 and 0 for answering “Yes” and “No” or “Unclear” to each question, respectively. In this study, scores of 0–3, 4–7, and 8–11 indicated low, moderate, and high quality, respectively^49^. Upon evaluating the quality of the article, the two authors (TG and TK) individually assessed the articles. In cases of disagreement, a discussion was held with a third examiner (SF) to reach a consensus.

### Statistical analysis

The extracted articles and collected data were qualitatively integrated using tables and quantitatively integrated using a meta-analysis. For the meta-analysis of the impact of social frailty on all-cause mortality and functional disability, we used a random effects model, calculated the hazard or odds ratio with a 95% CI, and created forest plots for data integration. The I^2^ statistic was calculated to assess the heterogeneity between the studies. The greater the heterogeneity, the higher the I^2^ value. In this study, a value of 75% or more and a *p* value < 0.05 were considered to indicate a high level of heterogeneity^50^. In cases where high heterogeneity was observed, a sensitivity analysis was performed to verify the robustness of the results. Meta-analysis was performed using Review Manager version 5.4 (Cochrane Tech., Troy, MI, USA).

### Supplementary Information

Supplementary Information 1.

Supplementary Information 2.

## Data Availability

The data that support the findings of this study are available from the corresponding author, upon reasonable request.
