# Supplementary material for: Social frailty as a predictor of all-cause mortality and functional disability: a systematic review and meta-analysis
Source: Sci Rep. 2024 Feb 10;14:3410. doi: 10.1038/s41598-024-53984-3 (PMC10858956; doi:10.1038/s41598-024-53984-3)
Supplement: Supplementary file 1 — Supplementary Information 1. [file 41598_2024_53984_MOESM1_ESM.pdf]

Study or Subgroup	log[Hazard Ratio]	SE	Weight	Hazard Ratio IV, Random, 95% CI	Year
Garre-Olmo J	0.9895	0.4998	0.0%	2.69 [1.01, 7.16]	2013
Ma L	1.1857	0.1403	0.0%	3.27 [2.49, 4.31]	2018
Gobbens RJJ	0.157	0.0925	0.0%	1.17 [0.98, 1.40]	2021
Jujo K	0.4253	0.2119	53.2%	1.53 [1.01, 2.32]	2021
Ono R	1.1725	0.4413	18.3%	3.23 [1.36, 7.67]	2021
Adachi T	0.3436	0.3359	28.5%	1.41 [0.73, 2.72]	2022
**Total (95% CI)**			**100.0%**	**1.71 [1.15, 2.56]**	

Heterogeneity: $Tau^2 = 0.03; Chi^2 = 2.66, df = 2 (P = 0.26); I^2 = 25\%$
Test for overall effect: $Z = 2.64$ ($P = 0.008$)

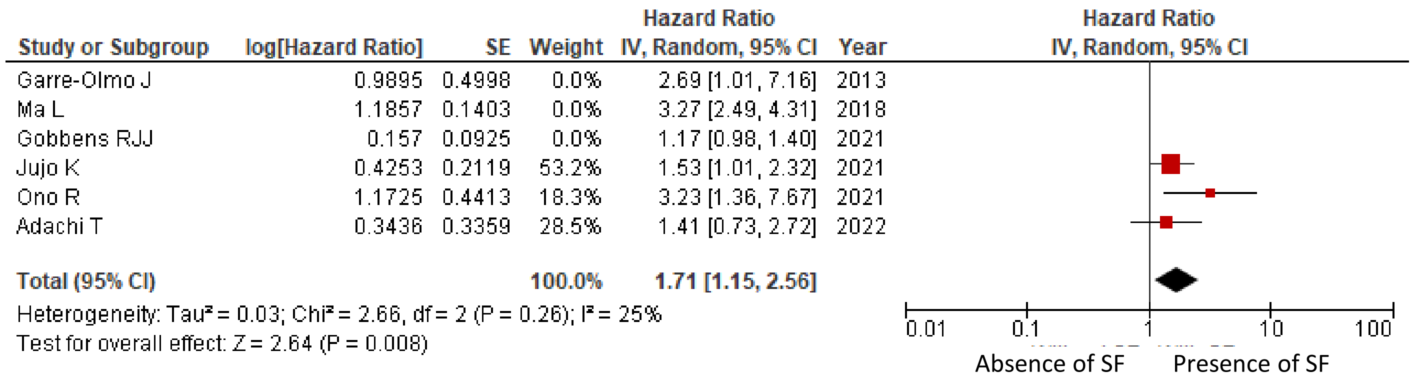

Supplementary File S1 Forest plot evaluating the effects of the presence or absence of social frailty on all-cause mortality.
