# Supplementary material for: Social frailty as a predictor of all-cause mortality and functional disability: a systematic review and meta-analysis
Source: Sci Rep. 2024 Feb 10;14:3410. doi: 10.1038/s41598-024-53984-3 (PMC10858956; doi:10.1038/s41598-024-53984-3)
Supplement: Supplementary file 2 — Supplementary Information 2. [file 41598_2024_53984_MOESM2_ESM.pdf]

Supplementary file S2 Results of quality assessment included studies.

Longitudinal studies

Author	Year	Selection				Comparability	Outcome		
		Representativeness of the exposed cohort	Selection of the non exposed cohort	Ascertainment of exposure	Demonstration that outcome of interest was not present at start of study	Comparability of cohorts on the basis of the design or analysis	Assessment of outcome	Was follow-up long enough for outcome to occur	Adequacy of follow-up of cohorts
Garre-Olmo J	2013	+	+	-	+	-	+	+	+
Ament BHL	2014	+	+	-	-	-	-	+	+
Makizako H	2015	+	+	-	+	++	+	+	+
Teo N	2017	+	+	-	+	++	-	+	+
Ma L	2018	+	+	-	+	+	+	+	-
Yamada M	2018	+	+	-	+	++	-	+	-
Gobbens RJJ	2021	+	+	-	+	+	+	+	+
Jujo K	2021	+	+	-	+	++	+	+	+
Ono R	2021	+	+	-	+	++	+	+	-
Adachi T	2022	+	+	+	+	++	-	+	-
Doi T	2022	+	+	-	-	++	+	+	+

Cross-sectional studies

Author	Year	Define source of information	List inclusion and exclusion criteria for exposed and unexposed subjects or refer to previous publications	Indicate time period used for identifying patients	Indicate whether or not subjects were consecutive if not population-based	Indicate if evaluators of subjective components of study were masked to other aspects of the status of the participants	Describe any assessments undertaken for quality assurance purposes	Explain any patient exclusions from analysis	Describe how confounding was assessed and/or controlled	If applicable, explain how missing data were handled in the analysis	Summarize patient response rates and completeness of data collection	Clarify what follow-up, if any, was expected and the percentage of patients for which incomplete data or follow-up was obtained
Gobbens RJJ	2015	+	+	+	+	-	U	+	+	-	+	-
Teo N	2017	+	+	+	+	-	U	+	+	+	U	-
Park H	2019	+	+	+	+	-	U	+	+	+	U	+
Usui N	2021	+	+	+	+	-	U	+	+	-	U	-
